# Childhood asthma prevalence: cross-sectional record linkage study comparing parent-reported wheeze with general practitioner-recorded asthma diagnoses from primary care electronic health records in Wales

**DOI:** 10.1136/bmjresp-2017-000260

**Published:** 2018-01-08

**Authors:** Lucy J Griffiths, Ronan A Lyons, Amrita Bandyopadhyay, Karen S Tingay, Suzanne Walton, Mario Cortina-Borja, Ashley Akbari, Helen Bedford, Carol Dezateux

**Affiliations:** 1 Life Course Epidemiology and Biostatistics, UCL Great Ormond Street Institute of Child Health, London, UK; 2 Farr Institute, Swansea University Medical School, Swansea, UK; 3 Clinical Epidemiology, Nutrition and Biostatistics, UCL Great Ormond Street Institute of Child Health, London, UK; 4 Centre for Primary Care and Public Health, Barts and the London School of Medicine and Dentistry, Queen Mary University London, London, UK

**Keywords:** paediatric asthma, asthma epidemiology, asthma in primary care

## Abstract

**Introduction:**

Electronic health records (EHRs) are increasingly used to estimate the prevalence of childhood asthma. The relation of these estimates to those obtained from parent-reported wheezing suggestive of asthma is unclear. We hypothesised that parent-reported wheezing would be more prevalent than general practitioner (GP)-recorded asthma diagnoses in preschool-aged children.

**Methods:**

1529 of 1840 (83%) Millennium Cohort Study children registered with GPs in the Welsh Secure Anonymised Information Linkage databank were linked. Prevalences of parent-reported wheezing and GP-recorded asthma diagnoses in the previous 12 months were estimated, respectively, from parent report at ages 3, 5, 7 and 11 years, and from Read codes for asthma diagnoses and prescriptions based on GP EHRs over the same time period. Prevalences were weighted to account for clustered survey design and non-response. Cohen’s kappa statistics were used to assess agreement.

**Results:**

Parent-reported wheezing was more prevalent than GP-recorded asthma diagnoses at 3 and 5 years. Both diminished with age: by age 11, prevalences of parent-reported wheezing and GP-recorded asthma diagnosis were 12.9% (95% CI 10.6 to 15.4) and 10.9% (8.8 to 13.3), respectively (difference: 2% (−0.5 to 4.5)). Other GP-recorded respiratory diagnoses accounted for 45.7% (95% CI 37.7 to 53.9) and 44.8% (33.9 to 56.2) of the excess in parent-reported wheezing at ages 3 and 5 years, respectively.

**Conclusion:**

Parent-reported wheezing is more prevalent than GP-recorded asthma diagnoses in the preschool years, and this difference diminishes in primary school-aged children. Further research is needed to evaluate the implications of these differences for the characterisation of longitudinal childhood asthma phenotypes from EHRs.

## Introduction

There is increasing use of coded information from primary care and hospital electronic health records (EHRs) to estimate the frequency, onset, persistence, severity and outcomes of long-standing childhood conditions, including childhood asthma.^1–7^ Asthma is the most common long-term childhood medical condition in the UK, among the highest prevalence in children worldwide,^8^ with 1 in 11 children aged under 16 years receiving treatment in the UK. Between-country comparisons of asthma prevalence in early childhood, such as those reported by the International Study of Asthma and Allergies in Childhood (ISAAC) and planned by the Global Asthma Network,^9^ are based on parent-reported wheezing symptoms suggestive of asthma using validated and standardised questionnaires within cross-sectional surveys.

More recently, the potential contribution of EHRs to our understanding of asthma has been highlighted by their use in a comprehensive assessment of asthma epidemiology and health service use and costs in the UK.^10^ EHRs offer an alternative approach to estimating the burden of childhood asthma at a population level and changes over time in relation to environmental exposures and interventions. However, the relation of epidemiological measures of disease frequency based on coded information recorded in general practitioner (GP) records to parent-reported wheezing suggestive of asthma across the early life course is unclear. Linking electronic health records within longitudinal studies such as birth cohorts offers an opportunity to compare the prevalence of parent-reported wheezing with that of asthma diagnoses derived from GP records across early childhood.

We compared GP-recorded asthma diagnoses documented in linked primary care EHRs to parent-reported wheezing over the first 11 years of life in Welsh children participating in the Millennium Cohort Study (MCS). We hypothesised that parent-reported wheezing in the preceding 12 months would be more prevalent than GP-recorded asthma diagnoses over the same time period, particularly among preschool-aged children, and examined the extent to which other respiratory diagnoses coded in GP records might account for any differences.

## Methods

### Subjects

The MCS is a prospective study of 18 819 British children born between 2000 and 2002 whose parents were first interviewed when their child was aged 9 months and subsequently at ages 3, 5, 7 and 11 years, when information was collected on social and health factors related to the child and their family. At the age 7 year interview, parents of 1840 (94.3%) of 1951 singleton children interviewed in Wales gave consent to link information collected within MCS to their child’s routine health records up to their 14th birthday.

### Linked records

Linkage of MCS data to routine primary care EHRs was facilitated by the privacy protecting Secure Anonymised Information Linkage (SAIL) databank, which currently has permission to use anonymised records for research from 78% of general practices in Wales. Datasets imported into SAIL are anonymised and linked by assigning unique Anonymised Linkage Fields to person-based records.^11^ Linkage was made to information in the Welsh Demographic Service for 1834 (99.7%) of the 1840 cohort members, 1529 (83.1%) of whom were registered before their 14th birthday with a GP participating in SAIL: 1501 (98.2%) of children registered with these practices had at least one GP event (diagnosis or prescription) record. At ages 3, 5, 7 and 11 years, respectively, 129, 101, 102 and 85 children were not registered with a GP during the relevant 12-month time period used to estimate period prevalence. The parents of 116, 76, 0 and 226 children did not take part at the age 3, 5, 7 and 11 year interviews, while across all ages, a total of 17 children had missing information on reported wheeze. Thus, linked records were available for 1280 (607 girls), 1347 (645 girls), 1426 (685 girls) and 1211 (573 girls) children at ages 3, 5, 7 and 11 years, respectively, and were used to estimate prevalences. There were no differences in child sex, mean interview age, household income poverty (<60% national median income) or maternal history of asthma for children with a linked GP record compared with those without (data not shown).

### Wheezing and asthma diagnoses

We derived, for each child, a binary variable for parent-reported wheezing (yes/no) in the 12 months preceding their actual date of MCS interview at ages 3, 5, 7 and 11 years from parental responses to the ISAAC^12^ standardised question ‘Has your child had wheezing or whistling in the chest in the last 12 months?’

At the MCS interviews held at ages 5, 7 and 11 years, parents were asked if their child took any medicines on a regular basis (defined as every day for 2 weeks or more) that were prescribed by a doctor or hospital. If so, the type of medicine was recorded and coded using British National Formulary codes. We examined parent-reported use of asthma-related drug treatments, including use of bronchodilators (British National Formulary (BNF) code: 3.1) and corticosteroids (BNF code 3.2).

Asthma diagnoses were derived for each child using Read codes employed in the UK Quality and Outcomes Framework to identify GP-recorded asthma diagnoses and/or relevant prescriptions issued for asthma-related medications for children registered with a GP for the full year or part of it (see online supplementary file: appendix).^13^ GP-recorded asthma diagnosis (yes/no) was assigned to each child for the 12 months preceding their actual date of MCS interview at each age as follows: (1) diagnostic codes for asthma and/or prescription issued for an asthma-related medication (‘GP-recorded asthma diagnosis’) or (2) no diagnostic code for asthma or asthma-related prescriptions issued (‘no GP-recorded asthma diagnosis’). Read codes for other respiratory illnesses (see online supplementary file: appendix) were also identified for the same time periods for children with parent-reported wheezing and for whom there was no GP-recorded asthma diagnosis in that time period.

10.1136/bmjresp-2017-000260.supp1 Supplementary file 1

### Statistical analyses

Survey and non-response weights at age 7 years were calculated to account for clustered sampling and attrition due to loss to follow-up and non-consent to linkage.^14^ We estimated weighted period prevalence of parent-reported wheezing and GP-recorded asthma diagnoses and calculated their difference (parent-reported minus GP-recorded) using the prtesti command in STATA. We calculated the weighted 12-month period prevalence of other respiratory conditions coded by GPs for children with parent-reported wheezing and no GP-recorded asthma diagnosis. We estimated at each age the percentage of children for whom there was agreement between parent-reported wheezing and GP-diagnosed asthma and calculated Cohen’s kappa statistics. We also examined parent-reported asthma medication use for children with no reported asthma or wheeze within the last 12 months in the MCS. All calculations were performed using STATA/SE V.13.0 (Stata Corp, Texas, USA) and the R language and environment for statistical computing and graphics V.3.3.3 (The R Foundation for Statistical Computing, Vienna, Austria).

## Results

The prevalence of parent-reported wheezing was highest at age 3 years and declined thereafter (table 1). The majority of children with parent-reported wheezing experienced recurrent wheeze in the relevant time period: for example, at age 3 years, 27.3%, 49.7% and 23.0% children with wheezing in the preceding 12 months were reported to have experienced one, between two and four, or five or more attacks, respectively, over the same period. The majority of children with parent-reported wheezing were also reported to have ever had eczema and/or hay fever, and this increased with age. At ages 3, 5, 7 and 11 years, the weighted percentage (95% CI) of eczema and/or hay fever was 58.4 (51.9 to 64.5), 61.8 (54.0 to 69.1), 69.8 (63.8 to 75.3) and 72.1 (65.2 to 78.1), respectively, among those with parent-reported wheezing in the preceding 12 months.

**Figure 1:**
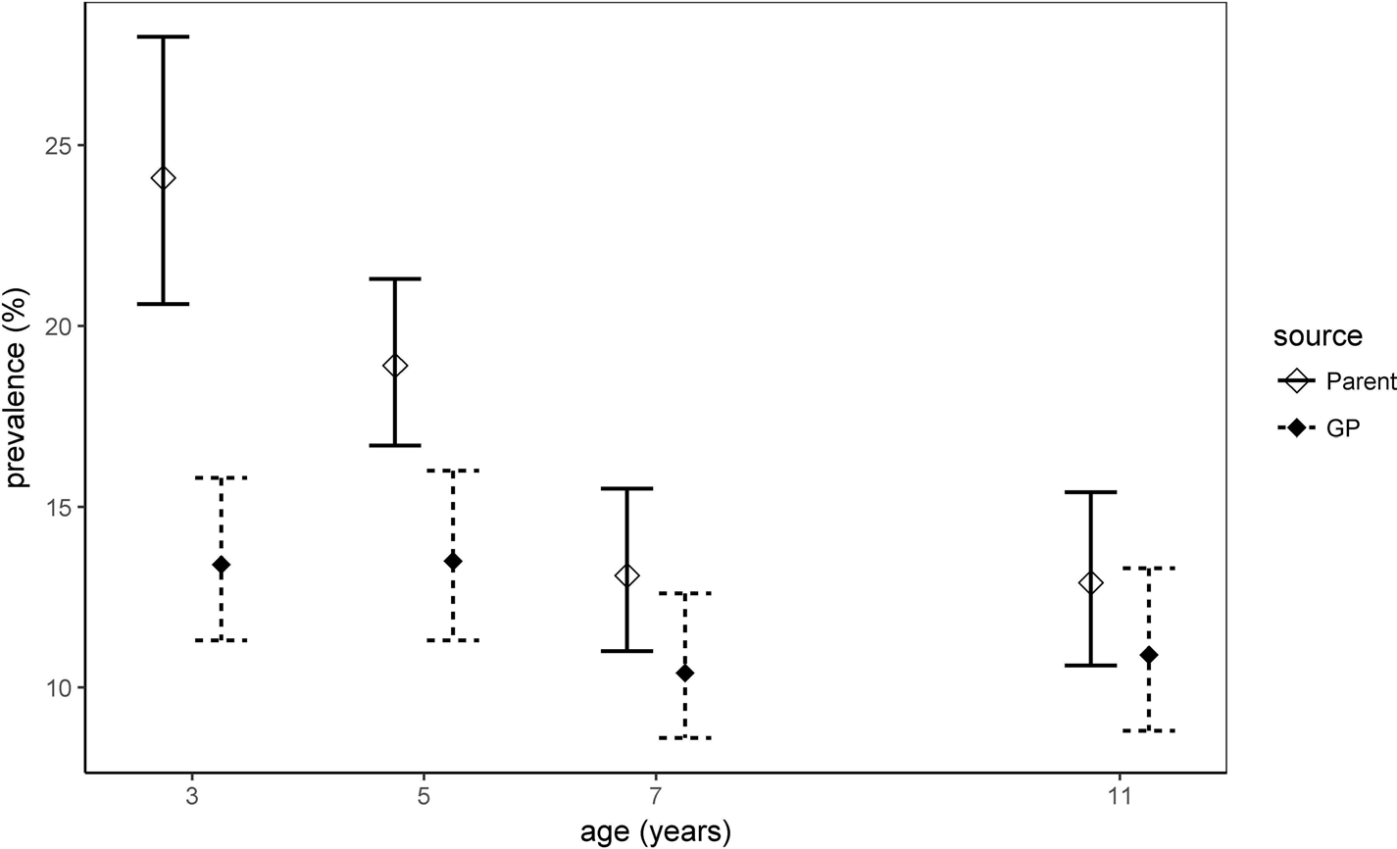
Weighted period prevalence of parent-reported wheezing and GP-recorded asthma diagnoses at specified ages. GP, general practitioner.

GP-recorded asthma diagnosis prevalence was lower on average than that of parent-reported wheeze and declined similarly, although less steeply, with age. The difference between these prevalences was greatest at ages 3 and 5 years and diminished with age such that by 11 years, differences were no longer significantly different (table 1 and figure 1).

**Table 1:** Weighted period prevalence (%), absolute difference and agreement between parent-reported wheezing and GP-diagnosed asthma in 12 months preceding each cohort interview

Target age for interview (years) n (n girls; w%*)	Child age at interview (years) Median (IQR)	Period prevalence of parent-reported wheezing % (95% CI) (A)	Period prevalence of GP-recorded asthma diagnosis % (95% CI) (B)	Absolute difference between parent-reported wheezing and GP-recorded asthma diagnosis† % (95% CI) (A–B)	Agreement (%) between parent-reported wheezing and GP-recorded asthma diagnosis % (95% CI)	Cohen’s kappa (95% CI)
Three years n=1280 (607; 46.0%)	3.1 (3.0 to 3.1)	24.1 (20.6 to 28.0)	13.4 (11.3 to 15.8)	10.7 (7.7 to 13.7)	80.8 (78.6 to 83.0)	0.39 (0.33 to 0.45)
Five years n=1347 (645; 46.5%)	5.3 (5.1 to 5.5)	18.9 (16.7 to 21.3)	13.5 (11.3 to 16.0)	5.4 (2.6 to 8.1)	85.8 (84.0 to 87.7)	0.50 (0.43 to 0.55)
Seven years n=1426 (685; 46.5%)	7.3 (7.1 to 7.5)	13.1 (11.0 to 15.5)	10.4 (8.6 to 12.6)	2.7 (0.3 to 5.1)	91.0 (89.5 to 92.4)	0.57 (0.50 to 0.63)
Eleven years n=1211 (573; 45.9%)	11.2 (11.0 to 11.5)	12.9 (10.6 to 15.4)	10.9 (8.8 to 13.3)	2.0 (−0.5 to 4.5)	91.8 (90.3 to 93.4)	0.61 (0.54 to 0.68)

*w%, weighted percentage.

†CI calculated using prtesti command in STATA on weighted prevalences. Similar estimates derived using McNemar’s test on unweighted prevalences (data not shown).

GP, general practitioner.

At each age, approximately 70% of parents of children with GP-recorded asthma diagnoses reported wheezing in their child over the same time period (table 2). A higher percentage of parents of children with no GP-recorded asthma diagnosis reported no wheezing, increasing from 83% at age 3 to 94.3% at age 11 years (table 2). Overall, agreement between parent-reported wheezing and GP-recorded asthma diagnoses increased with age, with Cohen’s kappa statistics suggesting moderate to substantial agreement by age 7 and 11 years (table 1). GP-recorded diagnoses of other respiratory conditions, predominantly of the upper respiratory tract, were identified in almost half of the children with parent-reported wheeze but no GP-recorded asthma diagnosis at 3 and 5 years (45.7% and 44.8%, respectively) and in almost one-third at 7 and 11 years (30.2% and 30.1%, respectively) (table 3). Of the parents who reported that their child did not have asthma and had not wheezed in the last 12 months, none reported regular use of asthma medications at ages 5 or 7 years, with only two reporting use at age 11 years.

**Table 2:** Parent-reported wheezing in 12 months preceding cohort interview compared with GP-recorded asthma diagnosis over same period by age

Parent-reported wheezing at interview	GP-recorded asthma diagnosis n (weighted %)		Overall
	Asthma	No asthma	
Age 3 years			
Yes	122 (70.4)	194 (17.0)	316 (24.1)
No	52 (29.6)	912 (83.0)	964 (75.9)
	174 (100)	1106 (100)	1280 (100)
Age 5 years			
Yes	127 (69.8)	132 (11.0)	259 (18.9)
No	59 (30.2)	1029 (89.0)	1088 (81.1)
	186 (100)	1161 (100)	1347 (100)
Age 7 years			
Yes	104 (72.4)	85 (6.2)	189 (13.1)
No	44 (27.7)	1193 (93.8)	1237 (86.9)
	148 (100)	1278 (100)	1426 (100)
Age 11 years			
Yes	95 (71.1)	59 (5.7)	154 (12.9)
No	40 (28.9)	1017 (94.3)	1057 (87.2)
	135 (100)	1076 (100)	1211 (100)

GP, general practitioner.

**Table 3:** Weighted period prevalence (%) of GP-recorded other respiratory diagnoses in 12 months preceding interview by age at cohort interview for children with parent-reported wheezing and without GP-recorded asthma diagnoses

Age at cohort interview	GP-recorded other respiratory diagnoses	
	Cases/total	Weighted % (95% CI)
Three years	88/194	45.7 (37.7 to 53.9)
Five years	59/132	44.8 (33.9 to 56.2)
Seven years	25/85	30.2 (21.9 to 39.9)
Eleven years	18/59	30.1 (20.0 to 42.5)

GP, general practitioner.

## Discussion

We found that both parent-reported wheezing and GP-recorded asthma diagnoses were more prevalent in the preschool years, with wheezing being significantly more prevalent than asthma diagnoses at these ages. By primary school age, there was moderate to substantial within-child agreement between the two measures. Parents of more than two-thirds of children with GP-recorded asthma diagnoses reported that their child wheezed in the preceding 12 months, and this percentage did not vary with age. Most parents of children without a GP-recorded asthma diagnosis reported that their child did not wheeze, and this percentage increased with age.

Our findings suggest that, at a population level, parent-reported wheezing prevalence is greater than GP-recorded asthma diagnosis prevalence in the preschool years, with smaller differences remaining in primary school-aged children. Differences in estimates of prevalence based on these two measures may arise for a number of reasons: parents may not take their child to the GP for wheezing, the GP may record a different diagnosis, or may not record any diagnosis, or parents may report other breath sounds as wheeze. Making a diagnosis of asthma in preschool-aged children who are generally unable to perform spirometry may be difficult. We found that other respiratory diagnoses, predominantly upper respiratory, were recorded in almost half of the preschool-aged children for whom there was no GP-recorded asthma diagnosis, lending some support for the second and fourth of these explanations. We also observed consistency between parental report of no wheezing and lack of parental-reported asthma medication use. Across all ages, around 30% of parents with a GP-recorded asthma diagnosis did not report wheezing in their child over the same 12-month period. While ISAAC questions selected a 12-month period for reporting to minimise recall bias, the possibility of such bias remains. Alternatively, asthma may be well controlled in some children with GP-recorded asthma diagnoses who may not have experienced any wheezing attacks, or children may have experienced symptoms other than wheeze, such as cough.

This is to our knowledge the first nationally representative longitudinal study to compare the prevalence of GP-recorded asthma diagnoses in EHRs with the prevalence of parent-reported wheezing across early childhood. In a smaller study using data from the Avon Longitudinal Study of Parents and Children, a birth cohort from South West England, Cornish *et al* linked 141 cohort members to their GP records and reported 67% agreement between ever-reported wheezing in the past 12 months and GP-recorded diagnosis of asthma by 9 years of age.^15^ Canova *et al* examined the GP records of 593 children born to mothers recruited in pregnancy to a longitudinal study of asthma: agreement between parent-reported symptoms or diagnoses and GP-coded diagnoses declined with age.^16^ Belgrave *et al* found comparable agreement between parental and physician ratings of wheeze in a regional birth cohort of 1184 children at ages 3, 5 and 8 years.^17^ Mukherjee *et al* reported the age-standardised annual prevalence of patient-reported symptoms suggestive of asthma to be 17.1% based on responses to national surveys using standardised questionnaires; this was significantly higher than the annual age-standardised prevalence of clinician-reported-and-diagnosed asthma (5.7%) and clinician-reported-diagnosed-and-treated asthma estimated from primary care EHRs.^10^ Direct comparison with our findings is not possible as these estimates were not based on comparisons of the same populations and were not presented for children separately.

To our knowledge, few other studies have examined the prevalence of childhood asthma in the UK using EHRs. Punekar and Sheikh^5^ estimated an 18-year period prevalence of clinician-diagnosed asthma in children and adolescents across the UK to be 22.9% (95% CI 22.3% to 23.4%) from practices contributing to the General Practice Research Database. In one study from the Netherlands, Pols *et al*^18^ estimated the prevalence of childhood asthma to be 6.1%, based on the requirement of at least two relevant consultations and at least two relevant prescriptions in the primary care record. This is closer to our estimate, which also included use of primary care-coded prescription as well as diagnosis.

Our estimate of the prevalence of parent-reported wheezing in the MCS 7-year-olds (13.1%) is lower than the prevalence of asthma symptoms (20.9%) in the UK reported by Asher *et al*^19^ in the ISAAC global Phase Three study, a cross-sectional questionnaire survey of 193 404 children aged 6–7 years from 37 countries. Kuehni *et al* reported the cross-sectional 12-month period prevalence of parent-reported wheeze in the Leicestershire longitudinal child cohort to be 12.4%, 12.5% and 20.5% at ages 3, 6 and 11 years, respectively, which is broadly consistent with our estimates.^20^ Arathimos *et al* analysed sex differences in parent-reported wheezing in MCS based on the ISAAC questionnaire and estimated the cross-sectional prevalence of wheeze in all MCS participants to be 14.4% in boys and 10% in girls at age 7 years.^21^

Strengths of our study include the use of a representative sample of Welsh children, high rates of consent and linkage, and adjustment for attrition and non-consent. We compared 12-month period prevalence of GP-recorded asthma diagnoses and parent-reported wheezing, allowing the age-specific relation between these measures to be assessed. Consistent and standardised definitions of wheezing and asthma diagnoses were employed at each age; however, parents were not asked to report clinician-diagnosed asthma and physiological measures of airway function were not available in our study.

It is widely acknowledged that asthma is a heterogeneous condition and that definitive diagnostic criteria for asthma are lacking; hence, we did not consider either measure as a gold standard. Validation of diagnoses recorded and coded within EHRs and the phenotypic algorithms used and assessment of their relation to existing accepted measures is needed to evaluate estimates of prevalence, causes and outcomes of chronic conditions such as childhood asthma based on EHRs.^22 23^ Parental understanding and interpretation of the term wheeze may impact on estimates of prevalence based on parent report.^24 25^ We found increasing agreement between parent-reported wheeze and GP-recorded asthma diagnosis with age. Our findings suggest that cross-sectional prevalence estimates of GP-recorded asthma diagnoses based on coded EHRs are likely to be lower than parent-reported estimates of wheeze in preschool-aged children. These differences become much smaller at later ages. Further research is needed to evaluate the implications of these cross-sectional differences in prevalence estimates among preschool-aged children for the characterisation of longitudinal childhood asthma phenotypes^21 26 27^ based on EHRs.
