# Supplementary material for: Childhood asthma prevalence: cross-sectional record linkage study comparing parent-reported wheeze with general practitioner-recorded asthma diagnoses from primary care electronic health records in Wales
Source: BMJ Open Respir Res. 2018 Jan 8;5(1):e000260. doi: 10.1136/bmjresp-2017-000260 (PMC5759709; doi:10.1136/bmjresp-2017-000260)
Supplement: Supplementary file 1 [file bmjresp-2017-000260supp001.pdf]

APPENDIX

Read codes[1] used to identify 'asthma diagnoses' in primary care electronic health records

Code	Description
Diagnoses	
H33..	Asthma (excluding H333.)
H3120.	Chronic asthmatic bronchitis
H3B..	Asthma-chronic obstructive pulmonary disease overlap syndrome
173A.	Exercise induced asthma
Asthma-related medications	
c1... – c15z.<br>c19..<br>c1B.. – c1EE.<br>c1c..	Selective Beta-Adrenoceptor Stimulant
c2... (excluding c23.., c24..)	Other Adrenoceptor Stimulants
c3... (excluding c32..)	Anticholinergic Bronchodilators
c4... (excluding c42.., c44..)	Xanthine Bronchodilators
c5... (excluding c52.., c51M., c51N.)	Compound Bronchodilators
c6...	Corticosteroids [Respiratory Use]
c7...	Asthma Prophylaxis
cA...	Leukotriene Receptor Antagonist
ck1..	Omalizumab

Read codes[1] used to identify 'other respiratory conditions' in primary care electronic health records

Code	Description
171..	Cough (no exclusions)
C370.	Cystic fibrosis (excluding C3700-C3701, C3703-C3709, C370y, C370z)
H0...	Acute respiratory infections (excluding H01.., H020., H021., H04.., H040., H0600-H0605, H0609, H060A, H0610-H0614, H07, H0y.., H0z..)
H2...	Pneumonia and influenza (excluding H203, H20y, H220., H221., H22y, H230, H232, H233, H23z, H24.., H25.., H26.., H27y, H27z, H28.., H29.., H2A, H2y..)
H30..	Recurrent wheezy bronchitis (no exclusions)
A33..	Whooping cough (no exclusions)
F512.	Glue ear (excluding F5121, F512z)
F52..	Suppurative and unspecified otitis media (excluding F5203, F521.-F524.)

[1] 5 byte version 2 Read codes obtained from NHS UK Read Codes Version 2 release 21.0.0_20160401000001 April 2016 combined terminology release
https://isd.digital.nhs.uk/trud3/user/guest/group/0/pack/9/subpack/21/releases
